# Quantitative changes in intracellular calcium and extracellular-regulated kinase activation measured in parallel in CHO cells stably expressing serotonin (5-HT) 5-HT_2A _or 5-HT_2C _receptors

**DOI:** 10.1186/1471-2202-13-25

**Published:** 2012-03-07

**Authors:** Patricia K Seitz, Nicole M Bremer, Andrew G McGinnis, Kathryn A Cunningham, Cheryl S Watson

**Affiliations:** 1 Center for Addiction Research, University of Texas Medical Branch, Galveston TX 77555, USA; 2 Department of Pharmacology and Toxicology, University of Texas Medical Branch, Galveston TX 77555, USA; 3 Department of Biochemistry and Molecular Biology, University of Texas Medical Branch, Galveston TX 77555, USA

**Keywords:** Serotonin, 5-HT_2A_R, 5-HT_2C_R, Intracellular calcium, pERK, Cell signaling

## Abstract

**Background:**

The serotonin (5-HT) 2A and 2C receptors (5-HT_2A_R and 5-HT_2C_R) are involved in a wide range of physiological and behavioral processes in the mammalian central and peripheral nervous systems. These receptors share a high degree of homology, have overlapping pharmacological profiles, and utilize many of the same and richly diverse second messenger signaling systems. We have developed quantitative assays for cells stably expressing these two receptors involving minimal cell sample manipulations that dramatically improve parallel assessments of two signaling responses: intracellular calcium (*Ca_i_*^++^) changes and activation (phosphorylation) of downstream kinases. Such profiles are needed to begin to understand the simultaneous contributions from the multiplicity of signaling cascades likely to be initiated by serotonergic ligands.

**Results:**

We optimized the *Ca_i_*^++ ^assay for stable cell lines expressing either 5-HT_2A_R or 5-HT_2C_R (including dye use and measurement parameters; cell density and serum requirements). We adapted a quantitative 96-well plate immunoassay for pERK in the same cell lines. Similar cell density optima and time courses were observed for 5-HT_2A_R- and 5-HT_2C_R-expressing cells in generating both types of signaling. Both cell lines also require serum-free preincubation for maximal agonist responses in the pERK assay. However, 5-HT_2A_R-expressing cells showed significant release of *Ca_i_*^++ ^in response to 5-HT stimulation even when preincubated in serum-replete medium, while the response was completely eliminated by serum in 5-HT_2C_R-expressing cells. Response to another serotonergic ligand (DOI) was eliminated by serum-replete preincubation in both cells lines.

**Conclusions:**

These data expand our knowledge of differences in ligand-stimulated signaling cascades between 5-HT_2A_R and 5-HT_2C_R. Our parallel assays can be applied to other cell and receptor systems for monitoring and dissecting concurrent signaling responses.

## Background

The serotonin (5-HT) 2A and 2C receptors (5-HT_2A_R and 5-HT_2c_R) are seven-transmembrane, G protein-coupled receptors (GPCRs) that are expressed in numerous brain regions. The 5-HT_2C_R protein is expressed predominantly in the central nervous system while the 5-HT_2A_R is also prominent in peripheral tissues, such as platelets and smooth muscle cells of the gut and vasculature [1]. Both receptors are involved in a wide range of physiological (e.g., temperature regulation, feeding) and psychological processes in mammals [2] and are implicated in psychological disorders (e.g., addiction, anxiety, depression, and learning and memory) [3-5]. These receptors share a high degree of homology, have overlapping pharmacological profiles, and utilize many of the same and richly diverse second messenger signaling systems. The most commonly studied downstream signaling pathway of the 5-HT_2A_R and 5-HT_2C_R is the activation of phospholipase Cβ (PLCβ) *via *G_αq/11 _proteins and the production of inositol-1,4,5-trisphosphate (IP_3_) and diacylglycerol (DAG), leading to increased Ca^++ ^release from intracellular stores [6,7]. However, both receptors also activate phospholipase A_2 _(PLA_2_), possibly through G_i/o _or G_12/13_, resulting in increased arachidonic acid (AA) release and have also been shown to activate phospholipase D [8,9] independent of PLC activation. The degree to which these downstream signaling pathways are recruited varies between the receptors, both in terms of the level of constitutive (agonist-independent) activation of each of the pathways [10] (particularly for the 5-HT_2C_R, where constitutive activity is highly dependent on the degree of editing) as well as ligand-directed signaling of agonists for the 5-HT_2A_R and 5-HT_2C_R [11]. These differences in signal activation may be a key feature distinguishing the functional effects of these two receptors. Thus, an appreciation of the full spectrum of downstream signal activation is critical when elucidating the functional actions of these receptors as well as in screening and evaluation of novel ligands for these receptors.

Parallel assays to measure simultaneous changes in IP_3 _and AA levels have been used to great advantage in deciphering differences in selective ligand-directed signaling [11], inverse agonism [12], desensitization [13,14], coupling specificity [15] and constitutive activity [16] between the 5-HT_2A_R and 5-HT_2c_R. However, additional rapid and quantitative assays to distinguish among further cellular responses in intact cells would broaden our appreciation of the multiplicity of signaling cascades likely to be initiated by serotonergic ligands. In the course of our studies to discover novel ligands for the 5-HT_2A_R and 5-HT_2C_R [17], we have developed quantitative live cell assays in parallel plates that involve minimal cell sample manipulations and improve measurements of signals leading to functional activity for cells expressing these two receptors. These assays measure two types of signaling evoked by ligand activation, changes in intracellular calcium (Ca_*i*_^++^) and phosphorylation changes in downstream kinases, in very similar cell preparations.

The assay to measure Ca_*i*_^++ ^levels utilizes detection of increased fluorescence in the presence of ionized calcium by the fluorescent dye Calcium 4 (Molecular Devices, Sunnyvale, CA) and is performed in live, attached cells, typically in 96-well plates [18]. Changes in Ca_*i*_^++ ^have long been recognized as critical to cell function, and techniques for measuring such changes have been rapidly evolving since the initial introduction of intracellular calcium-sensitive fluorescent dyes. We also developed an in situ immunoassay to detect activation of one class of the downstream mitogen-activated protein kinases (MAPKs), the extracellular-regulated kinases (ERK1 and ERK2), to measure an additional signaling event resulting from ligand binding. Phosphorylation of ERK is an example of an important integrator of upstream signaling events for many GPCRs as well as other cellular receptors [19-21], including the 5-HT_2A_R [22-27] and the 5-HT_2C_R [28,29]. At this pathway intersection many upstream signals are summed to subsequently coordinate actions leading to important cellular functions, such as cell survival/apoptosis [30], growth regulation [31,32] and differentiation [33]. Recently, activation of ERK was found to be inversely correlated with the degree of RNA editing of the 5-HT_2C_R; stimulation of the non-edited (as used in our studies) and partially edited isoforms caused greater levels of ERK activation than occurred with the fully edited version [34,35].

This assay to quantify the phosphorylated protein (pERK) uses phospho-specific antibodies and also is performed on attached cells fixed immediately following ligand treatment in 96-well plates [36,37]. ERK1/2 activation following 5-HT_2_R stimulation [28,34,38] has previously been demonstrated by measuring pERK with traditional methods, primarily densitometry of immunoblots. However, the plate immunoassay for pERK is a much more sensitive assay [37] and the 96-well format allows for far greater flexibility in assay design than traditional immunoblots. Many simultaneous experimental perturbations can be performed in the same cell preparation due to the rapid processing of high numbers of samples with these assays [36,37]. The assay also provides in situ detection of ERK activation as opposed to ELISAs and immunoblots that are performed on cell homogenates.

We developed these assays to compare the signaling effects resulting from stimulation by different serotonergic ligands in Chinese hamster ovary (CHO-K1) cell lines expressing either human (h)5-HT_2A_R or h5-HT_2C_R. These lines were developed by Berg and Clarke [39] and extensive data have been accumulated using the same lines in several laboratories [11,13-15,17,28,34]. However, it is necessary to optimize each assay for cell type and receptor system because access to signaling response compartments is different in cells of different tissues and lineages. In addition, technical aspects of measuring antigens (such as antibody concentrations and incubation times) vary for each protein. Moreover, values for these optimized parameters offer windows into the biological behavior of cells expressing different receptor subtypes. For example, the effects of cell crowding (density) on the downregulation of membrane receptors were demonstrated with this approach [40] as were the influences of culturing in serum (which itself contains many ligands). The power of the assays developed herein is the ability to rapidly determine multiple and subtle effects (potency, efficacy, second messenger activation) of various ligands with minimal cell disruption or comparison artifacts to yield important information concerning differences in signaling pathways triggered by activation in the 5-HT_2A_R- and the 5-HT_2C_R-CHO cells. Thus, interactions of second messenger systems with converging downstream enzymatic endpoints can be investigated.

## Methods

### Cell lines and cell culture

5-HT_2A_R-CHO and 5-HT_2C_R-CHO cells were a generous gift of K. Berg and W. Clarke (University of Texas Health Science Center, San Antonio). The FA4 line was transfected with h5-HT_2A_R (5-HT_2A_R-CHO cells) and the 1C19 line with unedited h5-HT_2C_R (5-HT_2C_R-CHO cells) in the p198-DHFR-Hygro vector containing a hygromycin resistance gene [39]. Reverse transcription of RNA followed by quantitative real time PCR assay for both transcripts confirmed that FA4 cells expressed high amounts of 5-HT_2A_R and no 5-HT_2C_R mRNA, that 1C19 cells expressed high amounts of 5-HT_2C_R and no 5-HT_2A_R mRNA, and that the parental cell line did not express detectable amounts of either mRNA (data not shown). Our recent data confirmed the lack of 5-HT_2C_R protein in FA4 cells and the lack of 5-HT_2A_R protein in 1C19 cells [41]. Receptor protein expression in both the FA4 and 1C19 cells has been assessed at 200 fmol/mg protein which approximates physiological levels in brain [39]. Cells were grown at 37°C, 5% CO_2 _and 85% relative humidity in GlutaMax™-MEM (Invitrogen, Carlsbad CA), 5% fetal bovine serum (Atlanta Biologicals, Atlanta GA), 100 μg/ml hygromycin (FA4 and 1C19, Mediatech, Manassas VA) or penicillin/streptomycin (parental cells, Invitrogen), and were passaged when they reached 80% confluence.

### Intracellular calcium assay

Changes in Ca_*i*_^++ ^levels were determined using the calcium-sensitive dye Calcium 4 (FLIPR No-wash kit, Molecular Devices, Sunnyvale CA, part #R8142). In our hands, the Molecular Devices kit produced results with reasonable signal intensity and reproducibility compared to other commercially available reagents (e.g., Fluo-3 and Fluo-4) without the necessity of dye removal or washing prior to measurements (data not shown). In addition to convenience, the lower number of manipulations required by this kit allowed for more rapid collection of data and decreased the likelihood of cell disturbance or loss.

Cells were plated in serum-replete medium at indicated densities in black-sided, clear bottom 96-well tissue culture plates. Care was taken to ensure even plating of cells, including frequent agitation or trituration of cells in the source reservoir. When plating volume was less than 150 μl, cells were less evenly distributed (visual observation; data not quantified), so we used 150 to 200 μl. Cells were added very slowly to the wells to minimize clustering around the edges. Placing the plate on a rotary shaker at low speed for several minutes following plating also helped to distribute cells evenly.

Except where indicated, cells were fed ~24 hrs later with serum-free medium. Following overnight incubation, medium was removed and replaced with 40 μl of fresh serum-free medium plus 40 μl of Calcium 4 dye solution supplemented with 2.5 mM water soluble probenicid (Invitrogen) to inhibit extracellular transport of the dye. Plates were returned to the 37°C incubator for 30-60 min then incubated for an additional 30-60 min at room temperature (RT) in the dark. Sixty-min incubations yielded higher levels of dye loading and higher relative fluorescence units (RFU) than 30-min incubations but did not appear to alter the final magnitude of the stimulations when expressed as percent baseline (data not shown).

Fluorescence (λ_ex _= 485 nm, λ_em _= 525 nm) was measured with a FlexStation3 (Molecular Devices). We used a larger number of measurements per well (eight), rather than the default value (six), and high detector sensitivity. A baseline was established for each well during the initial segment of each run. Addition of vehicle (Hank's balanced saline solution (HBSS), without CaCl_2 _or MgCl_2_) or 5x concentrated test substance occurred at 17 sec. The manufacturer suggests using no greater dilution than 5-fold because of issues with the rate of diffusion. We found that adding test solutions at faster speeds (up to speed 6 = 94 μl/sec) and using the trituration function (40 μl, 1-3 times) assisted rapid mixing and resulted in more reproducible data (data not shown). The CHO cells remained attached under these conditions; if using other cells lines, attachment should be monitored and reagent addition conditions modified as necessary.

Following addition of test reagent, fluorescence was recorded every 1.7 sec for 90-120 sec. For both 5-HT_2A_R and 5-HT_2C_R, the peak Ca_*i*_^++ ^response to agonists occurred 10 to 20 sec following stimulation. Maximum peak height was determined by the FlexStation software (SoftMax Pro 5.2) for each well. When testing inhibition by antagonists, 5x concentrated antagonist was added as above and the recording time shortened to 60 sec. This first round of measurements allowed us to measure any inherent agonist activity and also provided a 15 min antagonist preincubation period. Then vehicle or 5x concentrated agonist solution was added and a second round of measurements recorded inhibition of the agonist response.

Ca_*i*_^++ ^measurements were performed following both overnight (16-20-hr) and 48-hr incubations. The small difference in the magnitude of the fluorescent response was primarily due to increased cell numbers following the longer growth period and suggested that both time points were equally effective. Pre-coating of well surfaces with poly-D-lysine is a common method to improve attachment of cells during manipulations. We found no difference in the number of cells or in the fluorescent response between wells with and without 10 μg/ml poly-D-lysine pre-coating (data not shown), and consequently, wells were not coated with poly-D-lysine.

### Plate immunoassay for pERK

We adapted a previously developed plate immunoassay [36,37,42] to measure levels of pERK following receptor stimulation. Cells were plated at the densities indicated, grown for 24 hr in serum-replete medium then shifted overnight to serum-free medium; similar to results for the Ca_*i*_^++ ^assay, coating wells with poly-D-lysine had no effect on plate immunoassay results (data not shown) and therefore was not utilized. The day of the experiment, cells were fed with 80 μl of serum-free medium and returned to the incubator for 1-2 hrs, as adding medium alone caused a measurable activation of ERK that subsided by 1 hr (data not shown). Ligands were added as 20 μl of a 5x stock concentration for the indicated time. Reactions were stopped by the addition of 100 μl phosphate buffered saline (PBS; pH 7.4) with 4% paraformaldehyde (PFA; resulting in a final concentration of 2% PFA). Optimal fixation time for CHO cells in this assay was 45 min at RT (data not shown). Cells were then permeabilized with ice-cold methanol to ensure antibody access to intracellular antigens, washed with PBS, and blocked for 45 min at RT with 0.1% fish gelatin (Sigma). Cells then were incubated with 1:500 dilution of mouse monoclonal anti-pERK (p44/42, Cell Signaling, #9106) overnight at 4°C with gentle shaking. Background was determined in a group of eight wells incubated with no primary antibody. After washing with PBS, biotin-conjugated secondary antibody (Vector Labs, # BA-9200, 1:500 dilution in blocking solution) was added and incubated for 1 hr at RT. Following washing, alkaline phosphatase (AP) complexed with avidin (Vector Labs, #AK5000) was prepared according to the manufacturer's directions, added to the wells and incubated for 1 hr at RT. After washing, 50 μl the AP substrate para-nitrophenyl-phosphate (pNpp; Vector Labs, #SK-5900; 20 drops/10 ml) with levamisole (an inhibitor of endogenous phosphatases, Vector Labs, #SP-5000; two drops/10 ml) freshly prepared in 100 mM sodium bicarbonate was added and the plate was incubated at 37°C for 30 min. The absorbance of the yellow product para-nitrophenol (pNp) was measured at 405 nm (A_405_).

### Crystal violet staining

Data were normalized to total cell mass as measured by crystal violet staining, a value proportional to cell mass that can be used as an estimate of cell number in each well [43]. Wells were rinsed with water, air dried and 50 μl of crystal violet solution (0.1% in water) was added for 30 min at RT, and the wells were rinsed again. Cell-adsorbed dye was extracted by the addition of 50 μl of 10% acetic acid (30 min, RT) and absorbance read at 590 nm. Levels of pERK per well were expressed as A_405_/A_590_.

### Data analysis

Replicates of 3-8 wells were utilized for Ca_*i*_^++ ^assays and replicates of 5-8 wells for pERK assays. The number of independent experiments performed is indicted in individual figure legends. Differences between groups were determined by one-way ANOVA, followed by Bonferroni *post hoc *test; *p *values < 0.05 were considered significant.

### Reagents and ligands

5-HT (Acros Organics, NJ); 1-[2, 5-dimethoxy-4-iodophenyl]-2 aminopropane (DOI), crystal violet and poly-D-lysine (Sigma, St. Louis, MO); SB216641 and SB 242084 (Tocris Bioscience, Ellisville MO); racemic M100907 (synthesized [17] in the laboratory of Scott Gilbertson, University of Houston, Houston, TX)

## Results

### Intracellular calcium assay

#### Cell density

**Figure 1:**
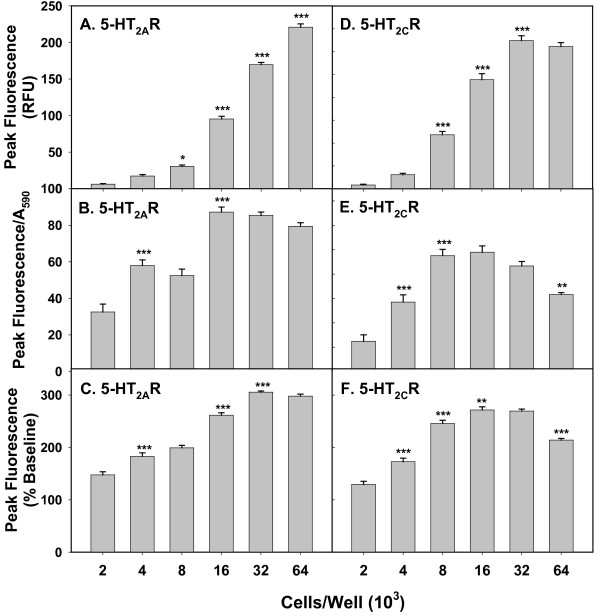
**Effect of plating density on Ca_*i*_^++ ^response to ligand stimulation of 5-HT_2A_R-CHO (A-C) and 5-HT_2C_R-CHO cells (D-F)**. Cells were plated at indicated densities, loaded with Calcium 4 and stimulated with 1 μM 5-HT. (**A, B**) Raw peak fluorescence, expressed as relative fluorescent units (RFUs). (**C, D**) Cell number was estimated by staining with crystal violet and fluorescence normalized to A_590_. (**E, F**) Fluorescence was normalized to baseline fluorescence of each individual well. Graph is representative of three independent experiments. n = 7-8; * p < 0.05, ** p < 0.01, *** p < 0.001 vs. previous bar.

Changes in Ca_*i*_^++ ^release in response to a fixed concentration (1 μM) of 5-HT were measured at increasing cell densities in 5-HT_2A_R-CHO (Figure 1, A-C) and 5-HT_2C_R-CHO cells (Figure 1, D-F). Figure 1A/1D plots peak fluorescence; as expected, this parameter increased significantly (each bar is compared to the previous bar) as a function of plating density. Data in Figure 1B/1E were normalized to cell mass by the crystal violet assay. Data in Figure 1C/1F were expressed as a percentage of baseline fluorescence determined for each individual well. This representation also is dependent on cell number, but depends on other factors as well, such as dye loading; therefore, one would not necessarily expect a perfectly linear relationship.

Plating at low densities (2000-8000 cells/well) resulted in low RFUs, and the response was not strictly proportional when normalized to cell number (Figure 1B/1E). At these plating densities, there were noticeable spaces between cells at the time of assay. This increased the likelihood that one or more of the detection measurements occurred at an area without cells, thus decreasing mean signal intensity and increasing relative error. The response per cell was constant over the range of 16,000 to 64,000 cells per well for 5-HT_2A_R-CHO cells and over 8000 to 32,000 cells per well for 5-HT_2C_R-CHO cells (no significant differences compared to previous bar). Cells plated at 64,000 cells/well sometimes began to detach following overnight incubation, an effect more pronounced in the 5-HT_2C_R-CHO cells. Consequently, normalized response levels did not increase in 5-HT_2A_R-CHO cells (Figure 1B and 1C) or began to decrease in 5-HT_2C_R-CHO cells (Figure 1E and 1F) when cells were plated at 64,000 cells/well. This effect was evident when data were normalized in either fashion. Thus, we chose plating densities of 16,000-32,000 cells/well for subsequent studies with both 5-HT_2A_R- and 5-HT_2C_R-CHO cells.

As mentioned above, expressing the data as a percentage of baseline fluorescence (Figure 1C/1F) achieved a less precise normalization to cell number than utilizing the crystal violet method (Figure 1B/1E) for different cell densities. However, the percentage of baseline calculation can be performed directly with the FlexStation software (SoftMax Pro 5.2) without additional manipulations. Therefore, this method was utilized when cell plating density was constant within a given experiment.

#### Protocol adaptations for our cell systems

The protocol from the Calcium 4 and FlexStation manufacturer recommended adding dye solution directly to serum-containing growth medium. We had several concerns about this protocol: 1) serum contains multiple growth-promoting agents, ligands and binding proteins for small molecules. The effects of these factors can alter responses (stimulatory or inhibitory) and complicate interpretation of the data; 2) after overnight (or longer) incubations, differential evaporation might affect the amount of growth medium remaining and this effect might vary among wells; as a result, adding fixed volumes of dye reagent and test solutions to the medium could result in variable and imprecise final concentrations. Therefore, we explored a variety of modifications to assess the impact of these problems and to achieve a consistent and reproducible protocol.

**Figure 2:**
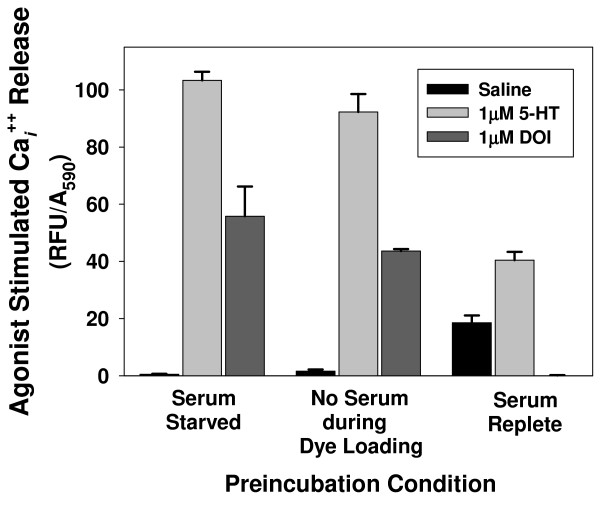
**Comparison of dye-loading protocols on stimulation by 5-HT and DOI in 5-HT_2A_R-CHO cells**. Cells plated at 16,000 cells/well two days prior to testing were either serum starved the day after plating ("Serum Starved"), serum starved only during dye loading ("No Serum during Dye Loading"), or serum replete for the entire experiment ("Serum Replete"). Calcium 4 dye reagent was added and cells were incubated for 60 min at 37°C then 60 min at RT. Cells were stimulated with a saline control medium (HBSS), 1 μM 5-HT or 1 μM DOI and peak fluorescence was recorded. n = 8 for saline and 3-4 for 5-HT and DOI.

**Figure 3:**
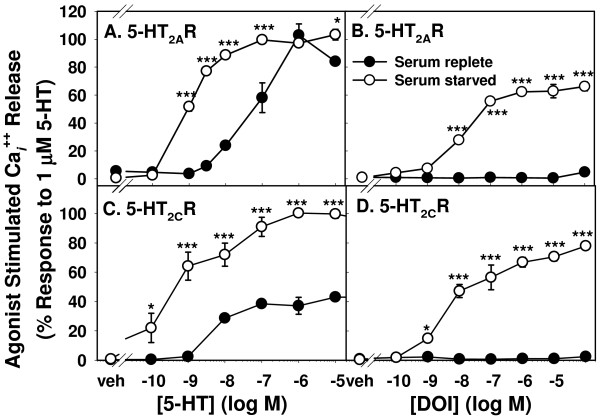
**Concentration and serum preincubation dependence of Ca_*i*_^++ ^release by 5-HT and DOI in CHO cells expressing 5-HT_2A_R (A, B) or 5-HT_2C_R (C, D)**. Cells plated at 16,000 cells/well in 96-well plates for 24 hrs were either preincubated overnight in serum-free (open circles) or in serum-replete (closed circles) medium and loaded with Calcium 4. Peak height following stimulation was recorded for each well and normalized to the maximal response to 5-HT. n = 5-6; * p < 0.05, *** p < 0.001 vs. respective serum preincubation.

The release of Ca_*i*_^++ ^evoked by 5-HT or the 5-HT_2 _agonist DOI was measured in 5-HT_2A_R-CHO cells to determine the effects of serum present during overnight preincubation and during Ca_*i*_^++ ^measurements (Figure 2). "Serum Starved" cells were preincubated overnight in *serum-free *medium, while "No Serum during Dye Loading" cells were preincubated overnight in *serum-replete *medium. Dye was loaded for these two groups by removing preincubation medium, adding equal volumes (80 μl) of fresh serum-free medium and dye then incubating as described in Methods. "Serum Replete" cells were preincubated overnight in medium containing serum; preincubation medium was *not *removed; 80 μl of dye was added directly to 80 μl serum-replete growth medium as per the manufacturer's instructions. Data in Figure 2 were normalized to crystal violet (A_590_) to account for the different cell numbers resulting from the different growth conditions. Addition of 1 μM of either 5-HT or DOI resulted in easily measurable fluorescence in both "Serum Starved" and "No Serum during Dye Loading" groups. When dye was added into the existing growth medium ("Serum Replete" cells), the response to 5-HT was dramatically reduced and the response to DOI was completely eliminated. In addition, vehicle (saline) alone produced a readily detectable response (18.6 +/- 2.6% compared to baseline) in the "Serum Replete" cells. The analogous experiment was not performed on 5-HT_2C_R-CHO cells because of the even more rigid requirement for serum-free preincubation in these cells: without serum starvation, there was no response to either 5-HT or DOI (see Figure 3C and 3D, below). Our purpose was to compare the protocol suggested by the manufacturer to protocols that utilized preincubation in serum-free conditions. The altered protocols were a clear improvement.

Therefore, we adopted a protocol that involved overnight preincubation in serum-free medium and replacement with fresh serum-free medium and dye solution prior to testing. Removal of the growth medium also enabled us to decrease the final volume in each well (80 μl as opposed to 160 μl) in subsequent experiments, thus doubling the number of samples that can be measured per bottle of dye and decreasing the expense for this costly reagent. This lower volume of reagent also allowed sufficient space in the wells for addition of the two reagents (antagonist and agonist) required in experiments measuring inhibition of Ca_*i*_^++ ^release by antagonists.

#### Effect of serum starvation

The concentration dependence of agonist-stimulated Ca_*i*_^++ ^release also was compared in cells preincubated overnight in serum-replete vs. serum-free media. Figure 3 shows Ca_*i*_^++ ^responses to varying concentrations of 5-HT (left panels) and DOI (right panels), all expressed as percent of the maximal response to 5-HT in serum-starved cells. Figure 3A, B show 5-HT_2A_R-CHO cells and Figure 3C, D show 5-HT_2C_R-CHO cells. Cells were incubated overnight in serum-containing (closed circles) or serum-free medium (open circles). In 5-HT_2A_R-CHO cells (Figure 3A), the 5-HT concentration response curve was shifted rightward by serum pretreatment, implying a decrease in ligand potency or cell sensitivity. However, the maximum response level was not significantly altered. In contrast, the 5-HT response in 5-HT_2C_R-CHO cells decreased in both potency/sensitivity and in maximum level of response (Figure 3C) when cells were pretreated with serum-replete medium. For both cell types, the maximal response to DOI was lower than the maximal response to 5-HT. The response to DOI was virtually eliminated with serum preincubation.

### Specificity of receptor responses

**Figure 4:**
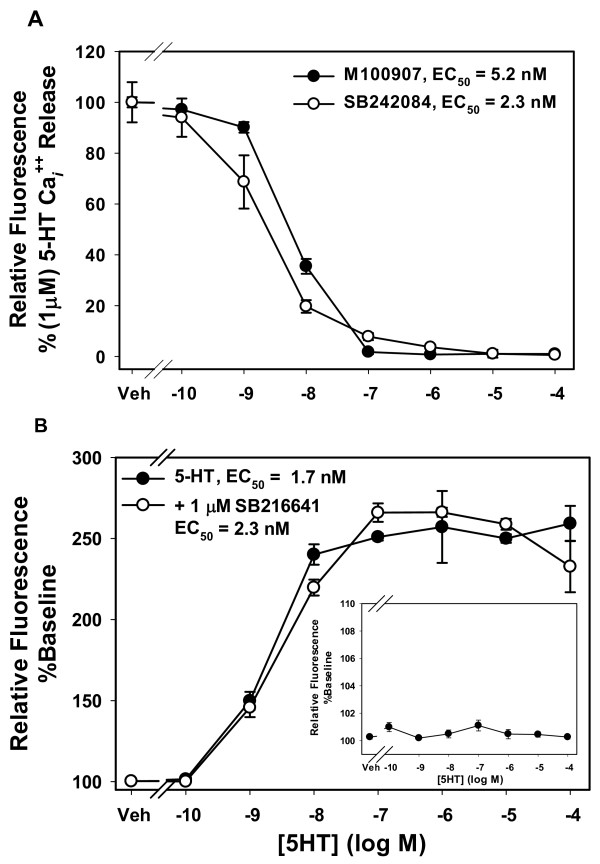
**Effect of specific antagonists on Ca_*i*_^++ ^release**. Cells were plated at 16,000 cells/well for 24 hrs, and loaded with Calcium 4 dye reagent. **A**. 5-HT_2A_R- or 5-HT_2C_R-CHO cells were preincubated with M100907 or SB242084, respectively, for 15 min then stimulated with 1 μM 5-HT. **B**. 5-HT_2C_R-CHO cells were preincubated with vehicle or 1 μM SB216641 for 15 min then stimulated with varying concentrations of 5-HT. **Inset**. Parental CHO-K1 cells (no preincubation) were treated with varying concentrations of 5-HT. n = 3-4 per group.

To confirm that the responses measured were the result of stimulation of the 5-HT_2A_R or 5-HT_2C_R in the respective cells lines, we measured inhibition of 5-HT-stimulated Ca_*i*_^++ ^release by selective antagonists. Figure 4A demonstrates that the Ca_*i*_^++ ^response was completely inhibited in 5-HT_2A_R-CHO cells by 15 min pretreatment with the selective 5-HT_2A_R antagonist M100907 and in 5-HT_2C_R-CHO cells by pretreatment with the selective 5-HT_2C_R antagonist SB242084. In addition, CHO-K1 cells have been reported to express 5-HT_1B_R. To ascertain whether the presence 5-HT_1B_R in the parental line contributed to response of the 5-HT_2A_R-CHO and 5-HT_2C_R-CHO cells, we used two approaches. Figure 4B (inset) shows no Ca_*i*_^++ ^release by the parental CHO-K1 cells to any concentration of 5-HT. (Note the expanded y-axis.) In a few experiments, these cells exhibited a small (< 5% above baseline) Ca_*i*_^++ ^release above 10^-6 ^M 5-HT but this was not a consistent finding. There was no detectable response to 5-HT in the pERK assay (data not shown). Figure 4B also depicts a Ca_*i*_^++ ^concentration response of 5-HT_2C_R-CHO cells to 5-HT alone and in the presence of 10^-6 ^M of the selective 5-HT_1B_R antagonist SB216641. There is no significant difference in the response at any concentration of 5-HT. Consequently, 5-HT_1B_R does not measurably contribute to the detected responses and SB216641 was not routinely included in subsequent assays.

#### Agonist concentration

Data in Figure 3 were used to determine EC_50_s for responses of the 5-HT_2A_R- and 5-HT_2C_R-CHO cells to 5-HT and DOI and are summarized in Table 1. DOI is usually considered a preferential 5-HT_2A_R agonist. However, DOI previously has been shown to significantly activate the 5-HT_2C_R in this cell line [11,28,34]. Responses to the preferential 5-HT_2C_R agonist MK212 were also measured following serum-replete and serum-free preincubation. There was no detectable Ca_*i*_^++ ^release in 5-HT_2A_R-CHO cells at any tested concentration up to 10^-4 ^M. Response to MK212 in serum-starved 5-HT_2C_R-CHO cells was of low magnitude (data not shown) and was greatly decreased when cells were preincubated in serum-replete medium.

**Table 1:** EC_50_values for agonist-stimulated Ca_*i*_^++ ^release

	5-HT_2A_R		5-HT_2C_R	
	**Serum-starved**	**Serum-replete**	**Serum-starved**	**Serum-replete**

**5-HT**	0.8 nM	53 nM	0.7 nM	4.9 nM

**DOI**	3.6 nM	> 24 μM	5.4 nM	> 100 μM

**MK212**	No response	No response	> 280 nm	> 11 μM

### Plate immunoassay for pERK

#### Time course of ERK activation

**Figure 5:**
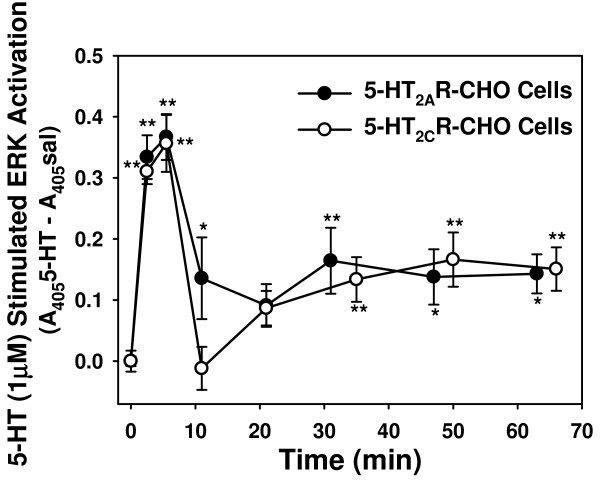
**Time course of ERK activation in 5-HT_2A_R- and 5-HT_2C_R-CHO cells**. Cells were plated at 16,000 cells/well for 24 hrs, preincubated in serum-free medium overnight and stimulated with vehicle (PBS) or 1 μM 5-HT for the indicated times. Levels of pERK were determined by plate immunoassay and net values were determined by subtraction of the vehicle control value from the 1 μM 5-HT value at each time point. Graph is representative of three independent experiments, n = 5-6/experiment; * p < 0.05, ** p < 0.01 vs. t = 0 min.

We measured the activation of ERK in 5-HT_2A_R- and 5-HT_2C_R-CHO cells at varying times. Cells (plated at 16,000 cells/well) were serum-starved overnight before treatment with 1 μM 5-HT for the indicated times (Figure 5). The time course of ERK activation was similar, though not identical, for these two cell lines. There was a distinct peak at 5 min followed by a return to baseline or near baseline levels of phosphorylation at 10 min. A second lower level increase was then sustained from 20 min to beyond 60 min, and was similar for both 5-HT_2A_R- and 5-HT_2C_R-CHO cells. Such a pattern is typical for ligand-stimulated ERK responses. The rapid post-activation dephosphorylation seen for the 5-HT_2C_R-CHO cells at 10 min is also a typical oscillating pattern [36,37,44-46]. A less dramatic and more variable drop was observed at 10 min for the 5-HT_2A_R-CHO cells, not reaching baseline until 20 min. A separate experiment examined more closely spaced time points and confirmed that there was no significant difference in responses in either 5-HT_2A_R-or 5-HT_2C_R-expressing CHO cells between 5 and 8 min (data not shown).

#### Cell density

**Figure 6:**
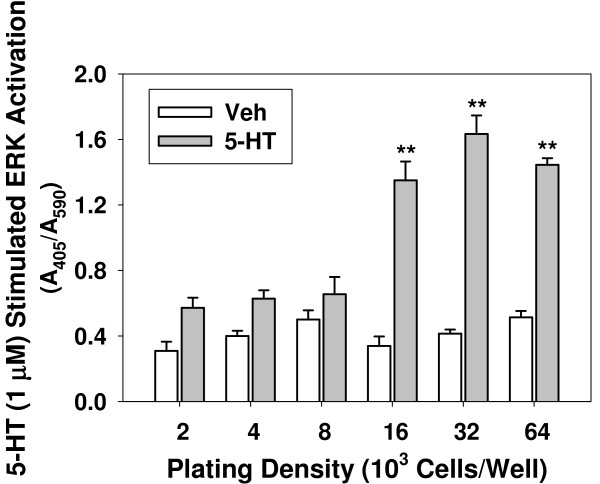
**Effect of plating density on ERK activation in 5-HT_2C_R-CHO cells**. Cells plated at the indicated densities for 24 hrs were preincubated overnight in serum-free medium, and stimulated with vehicle (Veh, open bars) or 1 μM 5-HT (gray bars) for 5 min. Levels of pERK were determined by plate immunoassay and normalized to cell number by crystal violet staining. Background (control wells without primary antibody) value was subtracted. Graph is representative of three independent experiments. n = 8; ** p < 0.01 vs. vehicle.

To determine whether cell density affects ERK phosphorylation of 5-HT_2_Rs expressed in CHO cells, we plated 5-HT_2C_R-CHO cells at densities between 2,000 and 64,000 cells/well and stimulated with varying doses of 5-HT for 5 min (Figure 6). Low cell numbers (< 8000 cells/well) yielded blunted 5-HT stimulations of pERK (on a per cell basis) over vehicle control, possibly for the same reasons as discussed for the Ca_*i*_^++ ^assay (above). Higher plating densities resulted in easily detectable levels of ERK activation that did not differ between 16,000 and 64,000 cells/well. Results for 5-HT_2A_R-CHO cells were similar (data not shown). Therefore, we adopted 16,000-32,000 cells/well as our standard cell plating density condition for ERK studies, as we did above for the Ca_*i*_^++ ^assays.

#### Dose response to agonists

**Figure 7:**
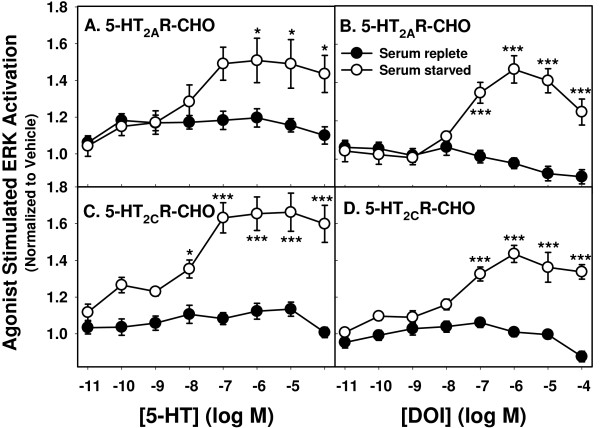
**Ligand concentration and serum preincubation dependence on ERK activation in 5-HT_2A_R- and 5-HT_2C_R-CHO cells**. 5-HT_2A_R- (**A **and **B**) and 5-HT_2C_R-CHO (**C **and **D**) cells plated at 16,000 cells/well for 24 hrs were preincubated in serum-replete medium (closed circles) or serum-free medium (open circles) overnight, followed by stimulation with vehicle (PBS) or indicated concentrations of 5-HT or DOI for 5 min. Levels of pERK were determined by plate immunoassay and normalized to cell number with crystal violet staining. Points represent data combined from three independent experiments (n = 5-6/experiment) normalized to vehicle control. * p < .05, ** p < 0.001 vs. vehicle.

We examined the activation of ERK by varying concentrations of the two ligands: 5-HT and DOI. Results (normalized to cell number) for 5-HT_2A_R-CHO cells are shown in Figure 7A, B and for 5-HT_2C_R-CHO cells in 7C, D. In both cell types, 5-HT and DOI caused concentration-dependent increases in pERK, with maximal responses at approximately 0.1 μM for 5-HT and 1 μM for DOI. EC_50 _values were: 12 nM (5-HT) and 31 nM (DOI) for 5-HT_2A_R; 14 nM (5-HT) and 19 nM (DOI) for 5-HT_2C_R. The magnitudes of the pERK responses to both DOI and 5-HT were similar in both 5-HT_2A_R- and 5-HT_2C_R-CHO cells.

#### Effect of serum starvation

We also compared the activation of ERK following overnight incubation in serum-replete (Figure 7, closed circles) versus serum-free (Figure 7, open circles) conditions (as described above for the Ca_*i*_^++ ^assays). Serum-replete preincubation clearly suppressed the response to both 5-HT and DOI in both 5-HT_2A_R- and 5-HT_2C_R-CHO cells.

## Discussion

Studies of cell signaling outcomes are complex, and the most-used techniques involve multiple and often different manipulations of the cells to achieve quantitative endpoints. The assays described here are minimally disruptive and offer improvements in speed, sensitivity, quantification and flexibility in assay design compared to traditional methods. For example, measurements of Ca_*i*_^++ ^release determined by fluorescent microscopy are time-consuming and yield data on either a small number of cells [45,47] or suspensions of potentially damaged cells. The No-Wash Ca_*i*_^++ ^protocol avoids multiple dye-removal and rinsing steps. Immunoblots are time consuming, and the protein extraction, gel electrophoresis and transfer steps introduce multiple sources of variability, making truly quantitative comparisons difficult or impossible. The assays described herein avoid these sources of error and provide easily quantifiable results while providing in situ information about concurrent signaling events.

### Temporal response patterns

For both 5-HT_2A_R and 5-HT_2C_R, the peak Ca_*i*_^++ ^response occurred 10 to 20 sec following stimulation, while activation of ERK was maximal at 5-8 min. These times agree with previously published data using similar methods [28] for these stably-transfected cells, and are consistent with sequential events in signaling pathways [48].

### Cell density

Higher numbers of cells provide greater membrane surface area, but different cell types and their receptors react differently to contact inhibition and/or the buildup of secreted cellular "factors" in the growth medium, sometimes resulting in decreased numbers of receptors/cell at higher cell densities [40,49]. For 5-HT_2A_R- or 5-HT_2C_R-CHO cells, cell density was not critical for either of the endpoints measured over a wide range, suggesting that contact inhibition of responses via these 5-HT receptors in CHO cells does not occur until the cells are extremely dense (> 64,000 cells/well). Differences in density-dependence likely hinge upon the individual receptor under study, the receptor family to which it belongs, the tissue of origin and the normal expression density. Also, receptors that are expressed naturally in a particular cell type may be subject to different control mechanisms than receptors that have been expressed in a heterologous cell type via transfection.

### Agonist concentration

The ligand concentration required to achieve maximal response varied between the two signaling measures. EC_50 _values for Ca_*i*_^++ ^stimulation were 1-3 nM while those for ERK activation were somewhat higher (Table 1 and Figure 7). This may suggest separate "wiring" for these two modes of signaling, or the contribution of Ca_*i*_^++ ^release to an ERK response that also requires the participation of other positive and/or negative signaling pathway components that were not the subject of our studies. While direct comparison of literature values of functional assays using different cell lines, different receptor expression levels, differently-edited isoforms (in the case of 5-HT_2C_R), and differing detection protocols is complex at best [50,51], the EC_50 _values for 5-HT- and DOI-induced Ca_*i*_^++ ^stimulation that we obtained are consistent with values obtained in similarly transfected 5-HT_2A_R- and 5-HT_2C_R-CHO cells [38] and in the same line of 5-HT_2C_R-CHO cells [28] using similar detection systems.

### Effect of serum starvation

Many cell surface receptors are down-regulated in the presence of serum [40]; overnight serum starvation is frequently used to minimize this effect. There was a notable difference in growth medium requirements between 5-HT_2A_R- and 5-HT_2C_R-expressing CHO cells with regard to the Ca_*i*_^++ ^response. In both, serum-free preincubation caused a leftward shift in the concentration response curves to 5-HT. Incubation of 5-HT_2C_R-CHO cells in serum-replete medium completely eliminated the response to DOI and markedly decreased the magnitude of the Ca_*i*_^++ ^response to 5-HT, while in 5-HT_2A_R-CHO cells, response magnitude was more variable. In 5-HT_2A_R-CHO cells, the level of response following the absence of serum during the two-hour dye incubation (Figure 2) suggests that two hours was sufficient to achieve Ca_*i*_^++ ^response levels similar to those seen with overnight serum starvation. Therefore, 5-HT_2A_R-CHO cells appear to be less affected by factors in serum than do 5-HT_2C_R-CHO cells, though serum inhibited the response to 5-HT- and DOI-induced stimulation of ERK activation in both cell types. A more proximal receptor-triggered event like Ca_*i*_^++ ^release may be more directly and differentially sensitive to serum-resident ligands like 5-HT. Serum can contain appreciable and variable levels of 5-HT and continuous exposure can result in desensitization [50]. In the case of the 5-HT_2C_R-CHO cells, the relatively high constitutive activity of the INI isoform may have resulted in a similar "ceiling effect", as could high levels of glutamate in the medium with serum-stimulated glutamate receptors leading to maximal influx of extracellular Ca^++ ^[52,53]. Exposure to such serum factors could result in responses with different temporal profiles for the two receptors, as ERK activation occurs as a result of many inputs and lengthy signal cascades, which may muffle the contributing effects of a single immediate response in the composite [36,37,44,45].

### Other signaling pathways

Although the best described pathway for 5-HT_2_R stimulation of ERK activation may be G protein and PLC-mediated Ca_*i*_^++ ^release, it is not the only route that has been documented. Distinct ligands for the same receptor can activate different pathways preferentially [11,36,37]. Differential coupling to PLCβ-mediated IP_3 _accumulation or to PLA_2_-mediated AA release has been particularly well studied in these 5-HT_2A_R-CHO cells [11,54]. The predominant choice or mixture of signaling mechanisms can also depend on receptor numbers and reserve [55][34], receptor conformation and occupancy [56] and prior exposure to agonists [50] or inverse agonists [12].

Other pathways have also been linked to ERK activation following ligand stimulation of 5-HT_2_R. In a variety of cell types, binding of calmodulin to the C-terminus of the 5-HT_2C_R recruits β-arrestin causes G protein-independent ERK stimulation [35,57-60]. Ligand stimulation of the 5-HT_2A_R can initiate a transactivation pathway by which epidermal growth factor (EGF) binds to its receptor (EGFR), resulting in ERK phosphorylation [20,27,61,62]. Stimulation of ERK that is partially independent of L-type Ca^++ ^channels and PLC has been described in arterial smooth muscle [22,23]. Thus, there are many different routes to ERK activation, and a more complete picture may emerge as we accumulate information in different cell types.

The presence of other receptors in these cells and the presence or lack of various other cellular components could also affect signaling cascades. In 5-HT_2_R-expressing HEK cells [38,57] low levels of endogenous G_αq/11 _[63] can complicate interpretation of results. Cross-talk among the 5-HT_2_Rs themselves [12,55,64] and with other families of co-expressed 5-HTRs [60][25,65] has been described. Further study of diverse ligands with alternative signaling endpoints should help to build the complex picture of integrated signaling in cells expressing this subset of 5-HT receptors.

### RNA editing

Finally, signaling by 5-HT_2C_Rs is also dependent on the population of edited receptor isoforms expressed [34,66], which affects their level of constitutive activity [34,67-69]. The partially and fully edited isoforms also demonstrate altered temporal and pharmacological characteristics for ERK activation, including pathway selection [34]. Primary cultures of mouse cortical neurons predominantly express constitutively active (less edited) isoforms [63] while the 5-HT_2C_R-CHO cells used here [11,14,39,64,70,71] and by Werry [28,34] were transfected with the unedited human 5-HT_2C_R isoform; other research has utilized partially-edited isoforms [38]. Therefore, differences between studies may be due to differences between natively-expressing vs. transfected cells and to the relative expression of edited and non-edited forms.

### Broader applications

The quantitative plate immunoassay is a particularly versatile assay that can be adapted to precisely measure a variety of important receptors and signaling protein activations and is limited only by the availability of sufficiently specific primary antibodies. With minor protocol modifications the assay has been used to quantify other cell surface receptors [40,42,72] the phosphorylation of other MAPKs (Jnk and p38) [44,73] and the dopamine transporter [74]. Relative intracellular vs. extracellular localization of receptors and other proteins can be detected by varying the fixation technique [49,72] and we have begun exploring applications to ex vivo tissue samples [75]. Differences in both Ca_*i*_^++ ^and MAPK signaling can be compared between many different cell types and transfectants.

## Conclusions

Serotonin- and 5-HT agonist-induced Ca_*i*_^++ ^release and ERK phosphorylation in 5-HT_2A_R- and 5-HT_2C_R-CHO cells showed many signaling characteristics that were similar, but with notable differences caused by growth media. Components in serum blunted the Ca_*i*_^++ ^response to 5-HT more dramatically in cells expressing 5-HT_2C_R than 5-HT_2A_R, and virtually eliminated the Ca_*i*_^++ ^response to DOI and pERK activation for both receptors. These studies show that measuring changes in Ca_*i*_^++ ^and ERK activation in parallel is a useful approach to dissecting intracellular responses to ligand activation. These quantitative, sensitive, and adaptable tools can be applied to a broad range of studies. The in situ, multiwell platform accommodates comparative data for multiple compounds within the same assay, which should facilitate the assessment of new drugs.

## Abbreviations

5-HT: serotonin; 5-HT_2A_R 5-HT_2C_R 5-HT_1B_R: 5-HT 2A, 2C and 1B receptors respectively; AA: arachidonic acid; *Ca_i_*^++^: intracellular calcium; CHO: Chinese hamster ovary cell line; DAG: diacylglycerol; DOI: 1-[2, 5-dimethoxy-4-iodophenyl]-2 aminopropane; EC_50_: 50% effective concentration; ERK: extracellular regulated kinase; GPCR: G protein-coupled receptor; HBSS: Hank's balanced saline solution; IC_50_: 50% inhibitory concentration; IP_3_: inositol-1,4,5-trisphosphate; MAPK: mitogen activated phosphokinase; PBS: phosphate buffered saline; pERK: phosphorylated ERK; PFA: paraformaldehyde; PLA_2_: phospholipase A_2_; PLCβ: phospholipase Cβ; pNp: para-nitrophenol; pNpp: pNp phosphate; RFU: relative fluorescence unit; RT: room temperature.

## Competing interests

The authors declare that they have no competing interests.

## Authors' contributions

PKS designed and performed experiments, analyzed data and drafted the manuscript. NMB and AGM carried out experiments and analyzed data. KAC and CSW conceived and oversaw the study, participated in experimental design and helped draft the manuscript. All authors read and approved the final manuscript.
